# Increase in the oxygen stable isotopic composition of water in wine with low ethanol yield

**DOI:** 10.1038/s41598-019-47331-0

**Published:** 2019-07-30

**Authors:** Fumikazu Akamatsu, Hideaki Shimizu, Aya Kamada, Yukari Igi, Tsutomu Fujii, Nami Goto-Yamamoto

**Affiliations:** 1 National Research Institute of Brewing, 3-7-1 Kagamiyama, Higashi-Hiroshima, Hiroshima, 739-0046 Japan; 2 Present Address: Faculty of Food and Agricultural Sciences, Fukushima University, 1 Kanayagawa, Fukushima, Fukushima, 960-1296 Japan

**Keywords:** Analytical chemistry, Element cycles, Stable isotope analysis, Applied microbiology

## Abstract

The stable isotopic composition of oxygen (*δ*^18^O) in wine is often analysed to determine the geographic origin of the wine and the amount of water dilution. However, little is known regarding the effects of two major winemaking techniques (the addition of acid (acidification) and sugar (chaptalization)) on the *δ*^18^O value of water in wine. Here we show that acidification and chaptalization have minor direct effects on the *δ*^18^O value but indirect effects based on the ethanol yield, which causes isotopic variation of up to 0.6‰. During fermentation, *δ*^18^O values increase at low ethanol yields, suggesting that yeast release water with a high *δ*^18^O value into wine when consuming sugars. Additionally, the ethanol yield is negatively correlated with the consumption of amino acids by the yeast, indicating that yeast growth decreases the ethanol yield. We therefore identify ethanol yield, which is decreased by the consumption of sugars by yeast for non-alcohol-fermentation processes as a potential factor leading to variations in the *δ*^18^O value of water during the winemaking process.

## Introduction

Measurement of the oxygen stable isotopic composition (*δ*^18^O) of water in wine is used for authentication, such as to determine the geographic origin of the wine and the extent of water dilution. These measurements compare data from the sample with data obtained for authentic products with the same origin^1,2^. The *δ*^18^O value of water in wine is similar to the *δ*^18^O value of water in the original grape must because the *δ*^18^O value of water is maintained as the water passes from the must to the wine during fermentation^3^. The *δ*^18^O values of water in grape berries are influenced by the *δ*^18^O value of the regional meteoric water^4^ as well as evapotranspiration^5^, soil water availability^6^, irrigation^7^, humidity^8^, and air temperature in the viticultural area^9,10^. Therefore, the *δ*^18^O values of water in wine have regional specific values^11,12^ and oxygen stable isotope analysis is thus used for discriminating geographical origins and detecting the watering down of wine^13–15^. However, little is known regarding the effects of two major winemaking techniques, namely, the addition of acid (acidification) or sugar (chaptalization), on the *δ*^18^O values of water during the winemaking process.

Here we test the hypothesis that acidification and chaptalization cause variation in the *δ*^18^O value of water in wine. Acidification involves adding an acid such as tartaric acid to prevent microbiological contamination and the production of off-flavours during fermentation, whereas chaptalization involves adding sugar to boost the final ethanol concentration in wine^16^. Acidification and chaptalization processes are controlled by the legal regulations of each winemaking area, and are commonly used when the grapes have low acid and sugar levels in order to meet the acid and sugar requirements for winemaking^17,18^. Understanding the effects of acidification and chaptalization on the *δ*^18^O value of water in wine will aid in authenticating and determining the geographical origin of the wine, and in enhancing its marketability.

## Results and Discussion

We tested this hypothesis by quantifying variations in the *δ*^18^O value of water in wine following fermentation during which acid or sugar was added to the wine (see Methods). After fermentation, we observed that the *δ*^18^O value of water in wine increased by 0.7 ± 0.2‰ (mean ± standard deviation, *n* = 40) from that of must (−6.4 ± <0.1‰, *n* = 20 for Cabernet Sauvignon and −6.4 ± <0.1‰, *n* = 20 for Chardonnay). This increase in the average *δ*^18^O value of water in wine compared to must was 0.8‰ higher than that reported previously^3^ and showed a relatively large variation of up to 0.6‰. Additionally, the *δ*^18^O value of water used to dilute concentrated wine grape juices in the same fermentation vessel did not differ significantly before (−7.8 ± <0.1‰, *n* = 10) and after (−7.8 ± 0.1‰, *n* = 10) the fermentation period (*t*-test, *t* = 0.69, *P* = 0.500, *n* = 20), suggesting that evaporation during fermentation has little impact on the *δ*^18^O value of water in wine.

We assessed the effects of acidification and chaptalization on the *δ*^18^O value of water in wine using general linear model (GLM): the treatment (control and acidification and/or chaptalization) and grape cultivar (Cabernet Sauvignon and Chardonnay) were set as fixed factors, the consumption of amino acids was set as a random factor to account for variation in yeast growth, and the ethanol yield was set as a covariate. We found no significant effect of either acidification or chaptalization on the *δ*^18^O values of water in both Cabernet Sauvignon and Chardonnay wines (Table 1), suggesting that these winemaking techniques would not directly affect the determination of the geographical origin of wine using oxygen stable isotope analysis.

**Table 1:** Results of GLM for the effects of treatment (acidification and chaptalization), grape cultivar (Cabernet Sauvignon and Chardonnay), and ethanol yield on the *δ*^18^O values of water in the wine.

	Control		Acidification		Chaptalization		Acidification and chaptalization		Treatment		Grape cultivar		Grape cultivar × treatment		Ethanol yield	
	CS	C	CS	C	CS	C	CS	C	*F*_3,30.47_	*P*	*F*_1, 29.98_	*P*	*F*_3,30.51_	*P*	*F*_1,31.00_	*P*
*δ*^18^O/‰	−5.7 ± <0.1^b,c^	−5.8 ± <0.1^c^	−5.5 ± 0.2^a,b^	−5.8 ± 0.1^c^	−5.5 ± 0.1^a^	−5.7 ± 0.1^b,c^	−5.5 ± 0.1^a,b^	−5.7 ± 0.2^a,bc^	2.27	0.100	14.20	<0.001	1.51	0.231	6.00	0.020

GLM was performed using treatment and grape cultivar as fixed factors, consumption of amino acids as a random factor, and ethanol yield as a covariate. *δ*^18^O values are presented as means and standard deviations (*n* = 5). Means with the same superscript are not significantly different (Tukey’s test). CS, Cabernet Sauvignon; C, Chardonnay.

**Figure 1:**
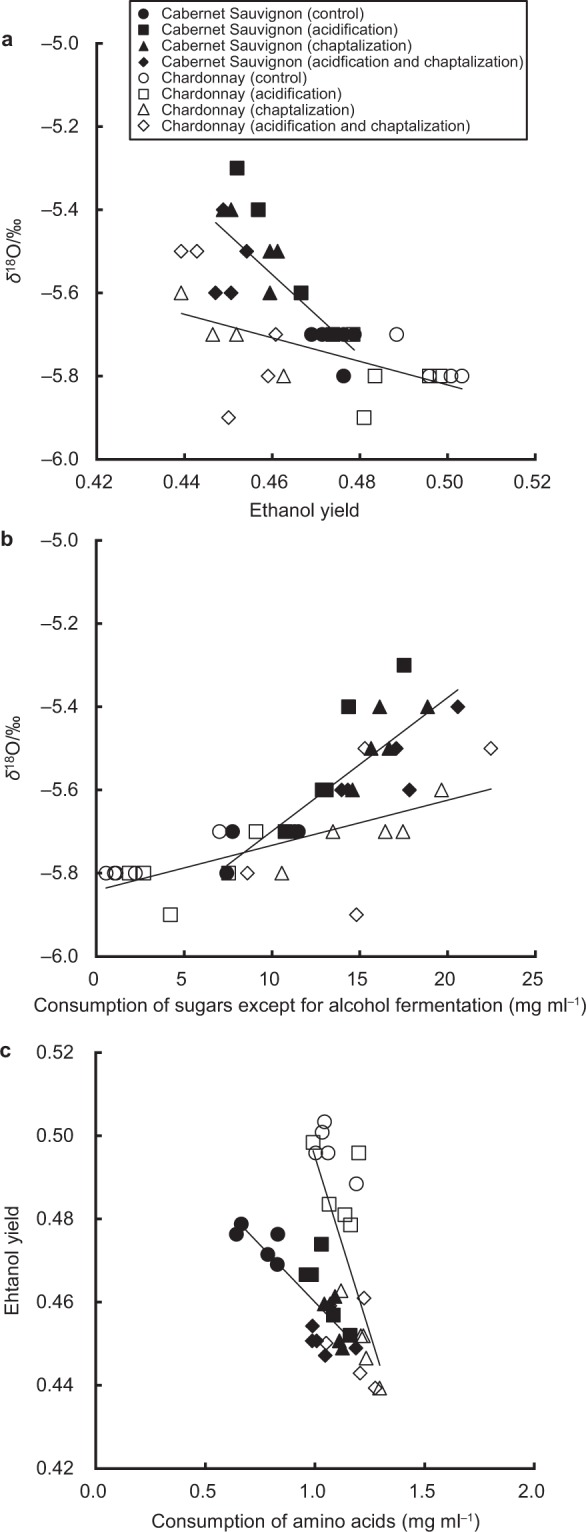
Variation in the *δ*^18^O values of water in Cabernet Sauvignon and Chardonnay wines during fermentation experiments. Relationship between (**a**) the ethanol yield and *δ*^18^O values of water, (**b**) consumption of sugars except for alcohol fermentation and the *δ*^18^O values of water in wine, and (**c**) consumption of amino acids and ethanol yield. The filled and open symbols represent Cabernet Sauvignon (*n* = 20) and Chardonnay (*n* = 20). Circle, square, triangle, and diamond symbols represent control (*n* = 5), acidification (*n* = 5), chaptalization (*n* = 5), and acidification and chaptalization treatments (*n* = 5), respectively, for each cultivar. The solid lines are the regression lines.

However, we found that the ethanol yield had a significant effect on the *δ*^18^O value of water in wine (Table 1), with the *δ*^18^O value negatively correlating with the ethanol yield of both Cabernet Sauvignon (linear regression, *R*^2^ = 0.58, *F*_1,18_ = 24.67, *P* < 0.001) and Chardonnay (linear regression, *R*^2^ = 0.35, *F*_1,18_ = 9.81, *P* = 0.006) (Fig. 1a). Additionally, we found significant effects of acidification and chaptalization on the ethanol yield (Table 2), suggesting that these winemaking techniques, particularly chaptalization may have indirect effects on the *δ*^18^O value of water in wine based on the ethanol yield. The theoretical maximum yield of ethanol is 51.1% in alcohol fermentation, while the actual ethanol yield was 46.6 ± 1.8% in both wines, which is about 5% less ethanol than the maximum (*n* = 40). The actual yield of ethanol did not reach the theoretical maximum because yeast incorporate glucose and fructose into biomass during fermentation^19^. The observed negative relationships suggest that the consumption of sugars by yeast for non-alcohol-fermentation processes such as biomass formation may be a principal factor leading to variations in the *δ*^18^O value of water during the winemaking process.

**Table 2:** Results of GLM for the effects of treatment (acidification and chaptalization) and grape cultivar (Cabernet Sauvignon and Chardonnay) on the ethanol yield in the wine.

	Control		Acidification		Chaptalization		Acidification and chaptalization		Treatment		Grape cultivar		Grape cultivar × treatment	
	CS	C	CS	C	CS	C	CS	C	*F*_3,31.50_	*P*	*F*_1,31.98_	*P*	*F*_3,31.33_	*P*
Ethanol yield	0.474 ± 0.004^b,c^	0.497 ± 0.006^a^	0.463 ± 0.009^c,d^	0.487 ± 0.009^a,b^	0.456 ± 0.006^d^	0.451 ± 0.009^d^	0.450 ± 0.003^d^	0.450 ± 0.010^d^	29.09	<0.001	37.30	<0.001	19.65	<0.001

GLM was performed using treatment and grape cultivar as fixed factors, consumption of amino acids as a random factor. Ethanol yields are presented as means and standard deviations (*n* = 5). Means with the same superscript are not significantly different (Tukey's test). CS, Cabernet Sauvignon; C, Chardonnay.

In support of this, we observed that the *δ*^18^O value positively correlated with the consumption of fructose and glucose by non-alcohol-fermentation processes in Cabernet Sauvignon (linear regression, *R*^2^ = 0.70, *F*_1,18_ = 42.48, *P* < 0.001) and Chardonnay (linear regression, *R*^2^ = 0.46, *F*_1,18_ = 15.14, *P* = 0.001) (Fig. 1b), but not with the consumption of sugars for alcohol fermentation in Cabernet Sauvignon (linear regression, *F*_1,18_ = 3.22, *P* = 0.090) and Chardonnay (linear regression, *F*_1,18_ = 2.55, *P* = 0.126). Generally, the *δ*^18^O values of carbohydrates in plants are higher than of the source water because the ^18^O enrichment of carbohydrate relative to source water is approximately + 27‰^5,20,21^. Additionally, atmospheric oxygen has a *δ*^18^O value of 23.5‰^22^ and the rate of isotopic exchange between oxygen and water is negligible at fermentation temperatures^23^; therefore the *δ*^18^O value of dissolved oxygen is expected to be the same as that of atmospheric oxygen^24^. In this study, the *δ*^18^O values of bulk sugars in must were 30.2‰ in Cabernet Sauvignon and 30.0‰ in Chardonnay, consistent with previous reports showing high *δ*^18^O values for carbohydrates in plants^25–27^. Therefore, we suggest that yeast respiration and propagation could significantly increase the *δ*^18^O values of water in wine due to the production of water with high *δ*^18^O values derived from dissolved oxygen and sugars in the must, respectively. Consequently, variations in the *δ*^18^O value of water during the winemaking process may be due to changes in ethanol yield relating to the consumption of sugars during respiration and growth under semi-aerobic conditions during the early stages of fermentation.

Additionally, the ethanol yield was negatively correlated with the consumption of amino acids by yeast in Cabernet Sauvignon (linear regression, *R*^2^ = 0.64, *F*_1,18_ = 32.35, *P* < 0.001) and Chardonnay (linear regression, *R*^2^ = 0.49, *F*_1,18_ = 17.00, *P* = 0.001) (Fig. 1c), suggesting that the consumption of amino acids by the growing yeast may contribute to the increase in *δ*^18^O values in wine. Many studies have reported that amino acids are used as a nitrogen source for yeast growth^28–30^. Our results showed that the consumption of amino acids was 1/10th the consumption of sugars except during the alcohol fermentation stage, suggesting that water generated from amino acids through nucleotide and protein metabolism pathways has a smaller impact on the *δ*^18^O value of water in wine than does water generated from sugar (on a stoichiometric basis). However, the consumption of amino acids would indirectly contribute to a high *δ*^18^O value because amino acids promote non-alcohol-fermentation processes through yeast growth.

In this study, we observed that acidification and chaptalization have minor direct effects on the *δ*^18^O value of water in wine but indirect effects based on the ethanol yield, depending on the consumption of sugars by yeast for the non-alcohol-fermentation processes. Further research is required to verify the effects of acidification and chaptalization on the yeast activities in terms of the *δ*^18^O value of water released from yeast during fermentation. Regardless, the present findings demonstrate quantitative identification of isotopic variation during the winemaking process and provide a new perspective on sugar consumption by yeast. Acidification and chaptalization may indirectly affect determination of the geographic origin of wine using oxygen stable isotope analysis. Further applications of this approach to other yeast groups, and to alcoholic beverages such as beer and sake, will enable testing of the generality of the present results. In addition, such further studies will aid in identifying other critical patterns in variations in the *δ*^18^O value of water in alcoholic beverages relating to yeast metabolism during the fermentation process.

## Methods

### Fermentation experiments

We conducted fermentation experiments following the acidification and chaptalization of two concentrated wine grape juices (Cabernet Sauvignon and Chardonnay, California Concentrate Company, CA, USA) each diluted 1:3 with the same water. Concentrated grape juice is uniformly aseptic, permitting specific detection of the effects of yeast on the *δ*^18^O value of water in wine. The Cabernet Sauvignon juice (must) contained 75.7 mg ml^−1^ glucose and 86.7 mg ml^−1^ fructose and the Chardonnay must contained 76.2 mg ml^−1^ glucose and 82.9 mg ml^−1^ fructose after dilution. Prior to fermentation, we treated the must samples in one of four ways: adding tartaric acid (1.6 mg ml^−1^) to reduce pH (<3.3) as an acidification treatment, adding sucrose (60 mg ml^−1^) to increase the ethanol content by 3% as a chaptalization treatment, adding both tartaric acid and sucrose as above for simultaneous acidification and chaptalization treatment, and adding nothing (control). Following treatment, we pre-activated the wine yeast (*Saccharomyces cerevisiae*, 100 mg ml^−1^, DV-10, Laffort Oenologie, Bordeaux, France) in dilution water at 37 °C for 20 min, added the yeast at 0.25 mg ml^−1^ final concentration to the must, then placed 40 ml aliquots of each sample in 50 ml conical tubes (Corning, Corning, NY, USA) with loose caps to allow the release of CO_2_ gas during fermentation. Samples were prepared in quintuplicate. We checked the effects of evaporation on the *δ*^18^O value during fermentation by adding 40 ml water samples (*n* = 10) to 50 ml conical tubes. All samples were fermented at 20 °C for 2 weeks.

### Chemical analysis

We measured the ethanol concentrations of the wine samples in duplicate using a 6890N gas chromatograph equipped with a split/splitless injector, a flame ionization detector (FID), and an auto sampler (Agilent Technologies, Santa Clara, CA, USA). The column was a DB-624 capillary column (30 m, 0.53 mm i.d., 3 μm film thickness; Agilent Technologies) and analysis was conducted according to the method previously described^31^. Briefly, each wine sample was diluted with 20 μl ml^−1^ 2-propanol aqueous solution (1:24, v/v; used as an internal standard), then 1.0 μl of sample was injected using split mode (1:40) at an injector temperature of 250 °C using helium as the carrier gas at a constant flow rate of 6 ml min^−1^. The column oven temperature was initially set at 50 °C and held for 5.5 min and the FID was held at 250 °C. Ethanol was quantified based on five-point linear normalization using external aqueous ethanol standards in the range 0–200 μl ml^−1^ (v/v). The ethanol concentration was expressed as ethanol content % (v/v).

The concentrations of glucose, fructose, and sucrose in the must and wine samples were determined using a Shimadzu HPLC system (Shimadzu Corporation, Kyoto, Japan) as described previously^32^. Aqueous acetonitrile (acetonitrile:water (3:1, v/v)) was used as the mobile phase. Samples were diluted with acetonitrile (7:3, v/v) and filtered through a 0.45-μm membrane, then 1 μl was injected into the HPLC system. Elution was carried out at a flow rate of 0.6 ml min^−1^ at 70 °C. We used the integrated concentrations of glucose, fructose, and sucrose as the total sugar concentration in each sample. We calculated the actual ethanol yield by dividing the concentrations of ethanol in the wine by the total sugar in the corresponding must. The consumption of sugar by the yeast except for non-alcohol-fermentation processes was defined as: (EtOH_t_ − EtOH_a_)/0.511) − Sugar_wine_, in which EtOH_t_, EtOH_a_, and Sugar_wine_ are the theoretical and actual ethanol concentrations and the residual sugar concentration in the wine, respectively. EtOH_t_ was calculated by: total sugar concentration in the must × theoretical ethanol yield (51.1%).

We determined the consumption of amino acids by the yeast by measuring the concentrations of amino acids and ammonium in the must and wine samples using a JLC-500/V amino acid analyser (JEOL, Tokyo, Japan). The consumption of amino acids by the yeast was calculated as the summation of differences between the concentrations of each amino acid in the must and corresponding wine samples.

### Oxygen stable isotope analysis

Sugars were purified by removing ionic and phenolic compounds from 0.5 ml must using solid phase exchange columns (OnGurard II A, H, and P, Dionex; Thermo Fisher Scientific, CA, USA) following a previously described protocol^33^. Aliquots of the purified sugars were injected into silver capsules, frozen, freeze-dried, then subjected to oxygen stable isotope analysis as described previously^27^. The *δ*^18^O values of the sugars were measured using a high temperature conversion/elemental analyzer coupled via a ConFlo IV universal interface to a Delta V isotope ratio mass spectrometer (Thermo Fisher Scientific). The *δ*^18^O values of water in the wine samples were also determined using a Delta V isotope ratio mass spectrometer and using the CO_2_ equilibration method according to the OIV-MA-AS2-12 method^34^. Samples were calibrated using three internal laboratory water standards (−14.5‰, −10.8‰, and −0.1‰) previously calibrated to the Vienna Standard Mean Ocean Water scale. Analytical errors (1 σ) for the standards were better than 0.2‰ over all analytical runs. Oxygen stable isotopic composition is expressed using small delta notation in parts per thousand, relative to the international Vienna Standard Mean Ocean Water standard.

### Statistical analysis

To evaluate the effects of acidification and chaptalization on the *δ*^18^O values of water or ethanol yield in wine samples, we performed general linear models (GLMs). In the models, we included the treatment (control and acidification and/or chaptalization) and grape cultivar (Cabernet Sauvignon and Chardonnay) as fixed factors and the consumption of amino acids as a random factor to account for variations in yeast growth. The interaction (grape cultivar × treatment) was included in the models. For *δ*^18^O values of water in wine, we included ethanol yield as covariate for the GLM. The means were separated using Tukey’s honestly significant difference test (Tukey’s HSD) after one-way analysis of variance. The relationships between the *δ*^18^O values of water in a wine, ethanol yield, consumption of sugar (except for non-alcohol-fermentation processes), and ethanol yield and consumption of amino acids and sugars except for alcohol fermentation were tested using Pearson’s product-moment correlation coefficient. Analyses were performed using JMP12.2 (SAS Institute Inc., Cary, NC, USA). For all tests, an α value of 0.05 was taken to indicate statistical significance (*P* < 0.05).
